# Determinants of Implant Utilization among Married Women of Childbearing Age in Chencha Town, Southern Ethiopia, 2017: A Case-Control Study

**DOI:** 10.1155/2020/4324382

**Published:** 2020-01-22

**Authors:** Rahel Abera, Mesfin Kote, Mulugeta Shegaze, Eshetu Andarge, Sultan Hussen

**Affiliations:** ^1^Department of Public Health, Arba Minch College of Health Sciences, P.O. Box: 155, Arba Minch, Ethiopia; ^2^Department of Public Health, College of Medicine and Health Sciences, Arba Minch University, P.O. Box: 021, Arba Minch, Ethiopia

## Abstract

Globally, 53% of women in reproductive age group use modern methods of contraception, with less than one percent of which using implants. In Ethiopia and other parts of sub-Saharan Africa, short-term contraceptive methods have been more utilized than long-acting methods like implants. Despite their effectiveness, implants have been underutilized due to various reasons. There is a dearth of stronger evidences on those factors in the country in general and the study area in particular. Therefore, this study aimed to identify determinants of implant utilization among married women of reproductive age at Chencha town, Gamo Gofa Zone, Southern Nations, Nationalities, and Peoples' Region (SNNPR) of Ethiopia. A community-based case-control study design was conducted among 324 women of reproductive age, 90 cases (users of implants) and 234 controls (users of short-acting contraceptives) from July to August 2017. Data were collected using a pretested, structured questionnaire through face-to-face interview. The data were entered and coded using Epi info 3.5.1 and then exported to Statistical Package for Social Sciences (SPSS) version 20 for cleaning and analysis. Descriptive analysis was done to quantify proportions, means, and standard deviations of variables. Bivariate and multivariable logistic regressions were done to identify the determinants of implant utilization. A total of 324 married women of reproductive age group were interviewed with response rate of 98%. In this study, the major determinants for implant utilization were desire to have 3–4 children {AOR = 0.104, 95% CI (0.03, 0.4)}, husband disproval {AOR = 0.11, 95% CI (0.038, 0.314)}, joint decision {AOR = 3.11, 95% CI (1.02, 9.48)}, and decision by other persons {AOR = 0.065, 95% CI (0.012, 0.352)}. This study found out that desire to have more children, husband disapproval, joint decision making, and decision by other persons were determinants of implant utilization among the target women. Implant utilization would improve through strengthening existing interventions targeting women with high fertility desire, transformation of gender norms in household decisions, and counseling for informed decisions.

## 1. Introduction

Implants are among the modern contraceptive methods that are hormonal, long-acting, and reversible contraceptives. They are small, thin, and flexible plastic rods that release a progestin hormone in the body, either levonorgestrel (Jadelle and Sino implant) or etonogestrel (Implanon). After being inserted under the skin of women's upper arm by trained health professionals, implants work by releasing a small amount of progestin hormone steadily into the blood that gives continuous protection for 3 to 5 years depending on the number of rods inserted [1–3].

Implants are one of the most effective reversible hormonal contraceptive methods ever developed as compared to short-acting methods. Overall, in three years of Implanon use, less than one pregnancy per 100 users can be expected, and this is 1.1 for the five years use of Jadelle and 0.9–1.06% cumulative pregnancy rate in the four years of use of Sino implant [4]. The use of long-acting methods like implants by couples in a country shows that the family planning (FP) program in that country is highly efficient in terms of management and financing in addition to being highly effective in spacing and limiting births. This is owing to implants' fewer requirements of providers and a lesser quantity of contraceptive supplies than a program relying on short-acting methods [5]. Furthermore, implants prevent unintended pregnancies, reduce the number of abortions, and lower the incidence of death and disability related to complications of pregnancy and childbirth [6]. Use of implants also frees a woman from using injectable contraceptives every three months and daily pills during the intended birth intervals because they are effective in preventing pregnancy from 3 to 5 years [5, 7].

Despite these benefits, the use of long-acting contraceptives (LACs) has not kept pace with that of short-acting methods, such as oral contraceptives and injectables. Data from demographic and health surveys (DHS) from four sub-Saharan countries show that the proportion of women currently using LACs is significantly lower than the proportion using short-acting methods. Globally, among women of reproductive age group, 53% use modern contraception methods but less than one percent use implants [8]. In many sub- Saharan African countries, fewer than 5% of women are using LACs [9]. The Ethiopian demographic and health survey of 2016 also showed that only 10% family planning (FP) clients utilized long-acting methods (8% implants and 2% IUCD) [1].

To respond to the low level of implant utilization, Ethiopia introduced community level implant insertion by trained health extension workers (HEWs). In order to strengthen and support the work of HEWs at the community level, the Ethiopia government introduced a new community mobilization structure called women development groups (WDGs) [8] which consists of a group of women networked in the villages where health extension workers visit and mobilize the candidate women for the appropriate Implanon uptake. Despite these efforts to improve the use of long-acting methods, the 2015 plan was not achieved [1].

Implants are relatively the preferred methods of contraception than other long-term family planning methods though not the commonest method in use in the country [10]. To find out the different factors that contribute to low utilization of implants in Ethiopia, there have been studies conducted on its prevalence and the factors associated with utilization [1, 3, 11–13]. Because of sociocultural variations in the different regions of the country, there is also variation in the factors affecting utilization of implants. Hence, there is a need for stronger evidences as studies employing stronger designs are lacking in the southern region of the country and in the study area in particular. Therefore, the present study tried to fill this gap through employing a case-control study among women of childbearing age in Chencha town.

## 2. Methods

### 2.1. Study Setting and Design

The study was conducted in Chencha town, Gamo Zone, South Ethiopia. The town is located 530 km to the southwest from Addis Ababa, the capital city of Ethiopia, and 298 km from the regional town Hawassa. The town is divided into 3 kebeles (the smallest administrative units in the Ethiopian government structure) and is situated at an altitude of 2732 metres above sea level. The total population of the town was 19,962, and of this, 10,141 were females and 9781 were males. There was one primary hospital offering healthcare services for the population of the town [14, 15]. A community-based case-control study was conducted among 324 women of reproductive age group (90 cases and 234 controls) from July to August 2017. Cases were married women of reproductive age group who have been using implants, whereas controls were married women of reproductive age group who have been using short-acting contraceptive methods (OCPs and injectables).

### 2.2. Sample Size and Sampling Techniques

The sample size was calculated using double population proportion using Epi info software version 7. Strongest determinants of implant utilization (i.e., income, husband approval, and educational status of women) were taken from previous research studies, and with this consideration, educational status of women gave the maximum sample size [3]. Hence, with a consideration of 5% level of significance ((*α*) = 0.05), 80% power (1-*β*), case to control ratio of 3 ((r) = 3), 7.9% education among controls (P1), and 19.5% education among cases (P2), the total sample size was calculated to be 300 (75 cases and 225 controls). Considering 10% nonresponse rate, the final sample size was found to be 330 (90 cases and 240 controls).

To select the samples, lists of the target women who were family planning users in the study period were obtained from family planning registries of health posts and Chencha Primary Hospital. A total of 115 implants users and 400 short-acting contraceptive users were available in the registration book, and the required samples of cases and controls were selected randomly from the list using select command in the SPSS software. Records were reviewed, and cases and controls selected were traced to their address through the guidance of HEWs.

### 2.3. Data Collection Procedure

The data were collected using a structured interviewer administered questionnaire, which was adapted from related literatures [4, 16]. The questionnaire was first prepared in English and translated to Amharic, the local language in urban areas of Southern Ethiopia, and again translated back to English to check for its original meaning. Before commencement of the actual data collection, pretest was done on 5% of the respondents in a kebele which was not included in the study, and the modifications on ambiguous words or items were done accordingly. Five nurses working in the surrounding district and a health officer were recruited for data collection and supervision of the data collection, respectively. A daylong vigorous training was provided to the data collection team on the objectives of the study, parts of the data collection instrument, and the study variables.

### 2.4. Measurement of Variables

The outcome variable was utilization of implants. In this study, *utilization of implants* is defined as when a woman has been either Implanon or Sino Implant or Jadelle user during the data collection period [4]. *Misconceptions about implants:* five questions were asked concerning misconceptions associated with implants. Those participants who scored above the mean on misconception items were categorized as having misconception and who scored the mean or below mean to misconception items were categorized as not having misconception.

### 2.5. Data Processing and Analysis

Data were entered and cleaned using Epi Info 3.5.1 and then exported to SPSS software version 20 for analysis. Bivariate and multivariable logistic regression analysis was conducted to identify determinants of implant utilization. Variables at *p* value of less than 0.25 in the bivariate logistic regression and prior strong significance were included in multivariable logistic regression analysis. Adjusted odds ratios with its 95% CI were used to determine the strength of association in the multivariable analysis. During the multivariable analysis, model fitness was checked using the Hosmer and Lemeshow goodness of fitness test. Multicollinearity among potential variables was checked using standard errors of less than 2 as a criteria for retention of the respective variable in the final model. Principal component analysis was conducted to set wealth index of participants. Level of statistical significance was declared at a *p* value of less than 0.05, and these variables were considered as determinants of implant utilization.

### 2.6. Ethical Considerations

The study was conducted after getting approval from the Institutional Review Board (IRB) in Arba Minch University.

## 3. Result

### 3.1. Sociodemographic and Socioeconomic Characteristics of the Study Participants

A total of 324 women participated in the study giving a response rate of 98%. The mean age (SD) of study subjects was 32 (±5.9) years. About 40% of cases and 38.9% of controls completed secondary and or preparatory education. Regarding household wealth of participants, 27 (30%) of cases and 55 (23.6%) of controls were in the lowest wealth quartile (Table 1).

### 3.2. Reproductive Characteristics of the Study Participants

About 86% of cases and 84% of controls married at age of 18 and above. Hundred percent of cases and controls had history of pregnancy and 8.9% of cases and 12.4% of controls had history of abortion. About 31 (34.4%) of cases and 94 (40.2%) of controls gave their first birth between 24 and 29 years of age. Nearly half of cases (52.2%) and controls (49.1%) had 1-2 children. Greater than 70% of cases and controls had a future plan to give birth (Table 2).

### 3.3. Contraceptive Use and Related Factors among the Study Participants

All the cases and controls heard about implants, and their main source of information was television for both cases and controls. All the cases and above 90% of controls mentioned implants and injectables as a method of contraception. About 77 (85.6%) cases had discussion with their husbands on implants, but 138 (59%) controls had no discussion on implants with their husbands. Majority of the cases (94.4%) and controls (85.9%) had discussion on implants with health professionals/HEWs. Majority of participants were not having misconception, 97.7% and 90.6% for cases and controls, respectively. All the cases and majority of the controls in the study were informed that implant was one of the FP options available in the health facilities. About 77 (85.6%) of cases and 196 (83.8%) of controls believed that health professionals keep their secret (Tables 3 and 4).

### 3.4. Determinants of Implant Utilization

In this study, the number of children desired was negatively associated with implant utilization. The odds of utilizing implants were 0.104 times lower for women who wanted to have 3–4 children than those women who wanted to have 1–2 children {AOR = 0.104, 95% CI (0.03, 0.4), *p* < 0.001}. This study also showed that women whose husbands disapproved the use of implant were having 0.11 times lower odds of using implants than those women whose husbands approved the use of implants {AOR = 0.11, 95% CI (0.038, 0.314), *p* < 0.000}. Another factor that showed significant association with implant utilization was decision making on contraception use. Women who had joint decision on contraception were having 3.11 times higher odds of using implants than those with self/husband decision only {AOR = 3.11, 95% CI (1.02, 9.48), *p* < 0.046}. Likewise, women who made the decision with the help of other persons like health professionals or friends on contraception use were having 0.06 times lower odds of using implants than those with self/husband decision{AOR = 0.065, 95% CI (0.012, 0.352), *p* < 0.002} (Table 5).

## 4. Discussion

The study findings showed that number of children desired for the future, husband approval, and decision on contraception use were found to be determinants for implant utilization among the study women.

Women who desired 3-4 children in the future were less likely to use implants compared with women who desired 1-2 children. This result was in line with study conducted at Debre Tabor town, North Gondar [17]. The possible explanation to this finding would be women's desire for fewer children may allow them to choose long-acting contraceptives while those planning to have more children tend to use short-acting contraceptives as they plan to give birth in short gaps. Besides, there is a common misconception that long-term family planning methods would delay fertility to the anticipated years of protection even if removed before method longevity. On top of this, such women might be of a higher degree parity [18] and prefer a longer period of spacing pregnancies than younger women who could likely use short-acting contraceptives. This could imply a gap in counseling women on the importance of no delay in return of fertility after implant use or the possibility of removing the implant at any time before the due date of removal.

In the study, women who reported that their husband disapproved their implant use were 89% less likely to use than their counterparts. A consistent finding was documented in previous studies in the country [12, 19–22]. Likewise, the study further revealed that joint decision on the method had significant association with utilization of implants. Women who decided with their husband jointly on the use of contraceptives were more likely to use implants than those women whose use was decided solely by the respective husband. This finding is similar to previous reports from different parts of Ethiopia and Zambia [4, 23, 24]. Studies conducted in different African countries including Ethiopia showed that involvement of women in household decision including fertility and choice of contraception had increased likelihood of modern contraceptive use by women [4, 23]. Since women respect gender norms in Ethiopia, they wait for approval of their husband, and husbands' opposition could either hinder or delay the decision to use a method. This calls for enhanced efforts in further transformation of gender norms as part of family planning programmes [25, 26]. Gender roles are in favor of men and limit women's autonomy in major family decisions, and the gender expectations can also limit the benefits that women are able to gain when they do decide to use family planning [27]. Thus, it strengthens evidences on the importance of male participation in family planning method choice and joint couple's decision on contraception to enhance the utilization [4, 23, 28].

Unlike joint decisions, decision made by other persons (health professionals or friends/relatives) on utilization of contraception resulted in lower odds of method use than decisions made by women themselves or their husbands. Though discussions with the healthcare provider or counseling improves uptake of long-acting family planning methods [4, 29–31], the decision has to remain the principal domain of either the woman or for the couple.

Even though the study brought stronger evidences on the determinants of implant utilization by employing a community-based case-control study, it has few limitations to consider. The study banked on participants' self-reported data, which was prone to recall bias due to the retrospective tracking of information. For reasons of resource scarcity, the data collectors were health workers from the nearby district; thus, a social desirability bias could also be evident.

## 5. Conclusion

High fertility desire, decision made by other persons (health care workers/friends/relatives), and husband disapproval negatively affected implant utilization while women having joint decision on contraceptive use had higher odds of implant utilization.

## Figures and Tables

**Table 1:** Sociodemographic and socioeconomic characteristics of women in Chencha town, Southern Ethiopia, August, 2017 (*n* = 324).

Variables	Cases *N* (%)	Controls *N* (%)	COR (95% CI)	*p* value
Age				
15–24	7 (7.8%)	21 (9%)	1	
25–34	54 (60%)	130 (55.6%)	1.25 (0.5, 3.1)	0.64
35 and above	29 (32.2%)	83 (35.5%)	1.05 (0.404, 2.72)	0.923

Women's education				
Cannot read and write	14 (15.6%)	41 (17.5%)	1	
Can read and write	5 (5.6%)	23 (9.8%)	0.64 (0.203, 1.99)	0.44
Primary school	10 (11%)	37 (15.7%)	0.792 (0.314, 1.997)	0.62
Secondary/preparatory	36 (40%)	91 (38.9%)	1.16 (0.564, 2.39)	0.69
College/university	25 (27.8%)	42 (17.9%)	1.743 (0.797, 3.814)^*∗*^	0.164

Husband's education				
Cannot read and write	4 (4.4%)	20 (8.5%)	1	
Can read and write	3 (3.3%)	24 (10.3%)	0.63 (0.125, 3.128)	0.567
Primary school	4 (4.4%)	22 (9.4%)	0.91 (0.2, 4.13)	0.902
Secondary/preparatory	10 (11.1%)	95 (40.6%)	0.526 (0.15, 1.85)	0.32
College/university	69 (76.7%)	73 (31.2%)	4.75 (1.54, 14.53)^*∗*^	0.007

Women's occupation				
House wife	43 (47.8%)	164 (70.1%)	1	
Merchant	9 (10%)	19 (8.1%)	1.81 (0.76, 4.28)	0.178
Government employee	35 (38.9%)	39 (16.7%)	3.42 (1.94, 6.032)^*∗*^	0.000
Other	3 (3.3%)	12 (5.1%)	0.953 (0.258, 3.53)	0.943

Husband's occupation				
Merchant	25 (27.8%)	66 (28.2%)	1	
Daily labourer	11 (12.2%)	39 (16.7%)	0.85 (0.8, 8.96)	0.89
Government employee	42 (46.7%)	74 (31.6%)	1.7 (0.72, 16.9)	0.65
Private worker	2 (2.2%)	17 (7.3%)	0.35 (0.024, 5.23)	0.45
Other	10 (11.1%)	38 (16.3%)	0.77 (0.71, 8.33)	0.83

Wealth index				
Very low	27 (30%)	55 (23.6%)	1	
Low	15 (16.7%)	61 (26.2%)	0.501 (0.242, 1.04)^*∗*^	0.063
Medium	31 (34.4%)	53 (22.7%)	1.2 (0.629, 2.26)	0.591
High	17 (18.9%)	64 (27.5%)	0.54 (0.267, 1.096)^*∗*^	0.088

^*∗*^Candidate variables for multivariable analysis at *p* value ≤0.25.

**Table 2:** Reproductive characteristics of women in Chencha town, Southern Ethiopia, August, 2017 (*n* = 324).

Variables	Cases *N* (%)	Controls *N* (%)	COR in 95% CI	*p* value
Age at marriage				
<18	13 (14.4%)	38 (16.2%)	1	
18 and above	77 (85.6%)	196 (83.8%)	1.148 (0.58, 2.273)	0.691

Number of pregnancy				
1–3	67 (74.4%)	169 (72.2%)	1	
4–6	19 (21.2%)	50 (21.4%)	0.959 (0.526, 1.745)	0.89
7 and above	4 (4.4%)	15 (6.4%)	0.673 (0.215, 2.1)	0.495

Number of abortion				
1	6 (75%)	17 (58.6%)	1	
2 and above	2 (25%)	12 (41.4%)	0.472 (0.81, 2.752)	0.404

Age at first birth				
<18	9 (10%)	19 (8.1%)	1	
19–23	12 (13.3%)	27 (11.5%)	0.938 (0.33, 2.67)	0.905
24–29	31 (34.4%)	94 (40.2%)	0.696 (0.286, 1.697)	0.425
30 and above	38 (28.8%)	94 (40.2%)	0.853 (0.325, 2.054)	0.728

Number of children alive				
1-2	47 (52.2%)	115 (49.1%)	1	
3-4	36 (40%)	98 (41.9%)	0.899 (0.539, 1.498)	0.682
Greater than 4	7 (7.8%)	21 (9%)	0.816 (0.325, 2.047)	0.664

Future plan of fertility				
To space	64 (71.1%)	180 (76.9%)	1	
To limit birth	26 (28.9%)	54 (23.1%)	0.738 (0.427, 1.277)	0.278

Desired number of children				
1-2	55 (85.9%)	108 (60%)	1	
3-4	6 (9.4%)	63 (35%)	0.187 (0.076, 0.459)^*∗*^	0.000
5 and above	3 (4.7%)	9 (5%)	0.654 (0.523, 1.561)	0.717

^*∗*^Candidate variables for multivariable analysis at *p* value ≤0.25.

**Table 3:** Contraceptive use related factors of women in Chencha town, Southern Ethiopia, August, 2017 (*n* = 324).

Variables	Case *N* (%)	Controls *N* (%)	COR in 95% CI	*p* value
Discussion with husband				
Yes	77 (85.6%)	96 (41%)	8.514 (4.48, 16.19)^*∗*^	0.000
No	13 (14.4%)	138 (59%)	1	

Husband approval on implant use				
Approves	67 (87%)	29 (30.2%)	1	
Opposes (disapprove)	10 (13%)	67 (69.8%)	0.065 (0.029, 0.143)^*∗*^	0.000

Decision on contraceptive use				
Self/husband	34 (37.8%)	41 (17.5%)	1	
Jointly (both together)	41 (45.6%)	9 (3.8%)	5.493 (2.342, 12.89)^*∗*^	0.000
Other people (HP, friends, or neighbors)	15 (16.7%)	184 (78.6%)	0.098 (0.049, 0.197)^*∗*^	0.000

Discussion with HP/HEW				
Yes	85 (94.4%)	201 (85.9%)	2.791 (1.054, 7.393)^*∗*^	0.039
No	5 (5.6%)	33 (14.1%)	1	

Myths and misconceptions				
Misconceived	2 (2.2%)	22 (9.4%)	0.108 (0.014, 0.816)^*∗*^	0.31
Not misconceived	88 (97.7%)	212 (90.6%)	1	

^*∗*^Candidate variables for multivariable analysis at *p* value ≤0.25. HP/HEW, health professional/health extension worker.

**Table 4:** Health facility related factors of women in Chencha town, Southern Ethiopia, August, 2017 (*n* = 324).

Variables	Category	Cases (*n* = 90)		Controls (*n* = 234)	
		*N*	(%)	*N*	(%)
Heard about implants	Yes	90	100	229	97.9
	No	0	0	5	2.1

Informed about implants as an option of FP	Yes	90	100	219	94
	No	0	0	14	6

Availability of implant in health facility	Yes	90	100	219	93.6
	No	0	0	15	6.4

Informed about the duration implants protect against pregnancy (3–5 years)	Yes	90	100	205	87.6
	No	0	0	29	12.4

Believe health professionals keep confidentiality	Yes	77	85.6	196	83.8
	No	13	14.4	38	16.2

**Table 5:** Predictors of implant utilization among (*n* = 324) women in Chencha town, 2017.

Variable	Cases *N* (%)	Controls *N* (%)	COR in 95% CI	AOR in 95% CI	*p* value
Desire number of children					
1-2	55 (85.9%)	108 (60%)	1	1	
3-4	6 (9.4%)	63 (35%)	0.187 (0.076, 0.46)^*∗*^	0.104 (0.03, 0.4)^*∗∗*^	0.001
5 and above	3 (4.7%)	9 (5%)	0.654 (0.523, 1.56)	1.51 (0.504, 4.52)	0. 46

Husband approval					
Approves	67 (87%)	29 (30.2%)	1	1	
Disapprove	10 (13%)	67 (69.8%)	0.065 (0.03, 0.14)^*∗*^	0.11 (0.04, 0.31)^*∗∗*^	0.000

Decision on method					
Self/husband	34 (37.8%)	41 (17.5%)	1	1	
Jointly	41 (45.6%)	9 (3.8%)	5.49 (2.34, 12.89)^*∗*^	3.11 (1.02, 9.48)^*∗∗*^	0.04
Other peoples	15 (16.7%)	184 (78.6%)	0.098 (0.05, 0.197)^*∗*^	0.065 (0.01, 0.35)^*∗∗*^	0.002

^*∗∗*^Statistically significant at *p* < 0.05 in multivariable logistic regression; 1 = used as reference category.

## Data Availability

The data used to support the findings of this study are available from the corresponding author upon request.

## Abbreviations

AOR: Adjusted odds ratio
CI: Confidence interval
COR: Crude odds ratio
FP: Family planning
HEW: Health extension worker
IRB: Institutional review board
LACs: Long-acting contraceptives
OCPs: Oral contraceptive pills
SNNPR: Southern Nations, Nationalities, and Peoples' Region
SPSS: Statistical Package for Social Sciences
WDGs: Women development groups
WHO: World Health Organization

## Additional Points

*Recommendation*. Healthcare providers providing family planning service need to target women with high fertility desires so that they can reduce their fertility intention by using long-acting family planning methods like Implanon. The local health office has to strengthen its capacity building activities so that women can get the right counseling to upshot free and informed decisions. The concerned policy makers in different sectors and levels of both government and nongovernmental organizations have to strengthen the transformation of gender norms in household decisions so that women can autonomously exercise their reproductive rights like making a free choice of contraceptives.
